# Innovative approaches to the study of social phenotypes in neurodevelopmental disorders: an introduction to the research topic

**DOI:** 10.3389/fpsyg.2013.00747

**Published:** 2013-10-17

**Authors:** Daniela Plesa Skwerer, Helen Tager-Flusberg

**Affiliations:** Department of Psychology, Boston UniversityBoston, MA, USA

**Keywords:** neurodevelopmental disorders, social phenotypes, cross-syndrome and longitudinal approaches, autism spectrum disorders, Williams syndrome

The field of social-affective neuroscience is growing exponentially, fueled by the availability and widespread use of non-invasive neuroimaging techniques, by advances in molecular genetics and by increasing sophistication in the behavioral characterization of social-affective functioning in both typical and atypical human development. In this context there has been a surge of interest in studying neurodevelopmental disorders (NDDs) as possible windows into the neurogenetic basis of the “social mind”. While holding great promise for advancing our understanding of genotype-phenotype relationships, research on neurogenetic disorders has encountered considerable challenges, ranging from the rarity of many NDDs with known genetic etiology and the considerable heterogeneity in phenotypic expressions within syndromes, to the difficulty of designing studies able to take in account the critical role played by developmental and epigenetic processes in shaping phenotypic outcomes. The articles collected in this e-book illustrate several ways in which researchers who study people with NDDs have attempted to overcome these limitations.

The studies selected—both reviews and original research articles—involve populations with NDDs whose phenotypes include core features related to social behavior, primarily autism spectrum disorders (ASD) and Williams syndrome (WS). We chose to focus on these NDDs because they have been viewed as opposite extremes on the social spectrum, and are often still portrayed this way in the popular media. Research findings, by contrast, reveal a much more complex mixture of social-affective strengths and weaknesses in both syndromes, underscoring the need for detailed descriptions of social phenotypes. This ebook brings together a representative sample of research approaches that have begun to uncover these complexities.

In the opening article, Asada and Itakura (2011) review recent research on aspects of social functioning that have received considerable attention in studies of both ASD and WS: face and emotion processing, social cognition, social engagement/motivation, communicative skills and diagnostic assessment outcomes. They discuss behavioral similarities and differences between WS and ASD, their possible developmental origins, and argue for expanding cross-syndrome comparisons to examining the *developmental pathways* leading to distinctive social-affective functioning in ASD and in WS, and to exploring the neural correlates of social-behavioral phenotypes.

Currently, much more is known about the neural substrates of social-behavioral phenotypes in ASD than in WS, but recent neuroimaging studies involving adults with WS are starting to bridge this gap. Haas and Reiss (2012) provide an integrative review of behavioral and neuroimaging studies with WS individuals, discuss the current status of knowledge about the social brain in WS, suggest a framework for understanding how the social brain develops in WS, and propose combining different neuroimaging techniques (fMRI, DTI, functional connectivity analyses) with behavioral tasks and neuropsychological assessments in longitudinal studies as directions for future research.

The two reviews are followed by a selection of original research studies representing various methodological approaches to investigating social phenotypes in NDDs.

The first article by Karmiloff-Smith et al. (2012) presents two case-studies of children with partial genetic deletions in the WS critical region (WSCR)—which encompasses about 28 genes deleted from one copy of chromosome 7: a girl with 24 of 28 genes deleted in the WSCR, and a boy with the opposite profile, i.e., only 4 genes deleted at the telomeric end of the WSCR (including GTF2I, which has been implicated in elevated levels of sociability typical of WS, in prior research). Cases of patients with partial deletions provide unique opportunities to examine the potential contribution of specific genes in the critical region to the unusual social phenotype seen in WS. Results from a large battery of experimental socio-cognitive tasks and standardized assessments revealed a partial WS socio-cognitive profile in the girl, contrasting with a more autistic-like profile in the boy, suggesting that deletion of the telomeric genes alone (including GTF2I) cannot fully account for the hypersociability typical of individuals with WS, and that genetic contributions to phenotypic outcomes involve complex interactions between genes.

The study by Cornish et al. (2012) combines cross-syndrome and prospective-longitudinal designs in examining how attentional profiles impact early socio-cognitive learning in children with WS, children with Down syndrome (DS), and typically developing (TD). These researchers found a complex pattern of *differential relationships* across teacher-report measures of inattention/hyperactivity/social-behavioral profiles and measures of receptive vocabulary and literacy in the WS compared to the DS groups, both concurrently and after 12 months, demonstrating domain-specific and syndrome-specific influences of attention deficits on socio-cognitive outcomes that appear related to the different genetic abnormalities underlying DS and WS.

Socio-communicative abilities, essential components of social phenotypes, are the focus of two articles, one addressing syndrome-specificity in pragmatic language impairments and their possible relations to molecular genetic variation, the other examining within-syndrome concurrent and longitudinal relations among particular pragmatic language skills.

Losh et al. (2012) conducted detailed cross-syndrome comparisons of performance on measures of pragmatic language ability and socio-cognitive skills in language-matched boys with idiopathic autism, fragile X (FRX) syndrome with autism, FRX without autism, DS and TD. Their behavioral results differentiated children with FRX with and without autism in their performance on both types of measures, sharpening the definition of syndrome-specific social-behavioral profiles in FRX and autism. They further examined possible molecular-genetic correlates of pragmatic language and theory of mind in the group of boys with FXS (disorder caused by ‘silencing’ of the FMR1 gene), and found that performance on the behavioral measures was correlated with FMR1-related variation, providing evidence for a testable link between genotypic and phenotypic variation.

Using an individual-differences approach and longitudinal design to investigate relations among pragmatic abilities in children with WS across developmental time-points and in relation to expressive vocabulary, John et al. (2012) found that the ability to verbally contribute new information within a social interaction showed stability from preschool to school-age in children with WS, and that differences in this pragmatic skill were predicted by children's ability to engage in triadic joint attention. This is an intriguing finding in light of evidence for atypical relations between early joint attention ability and vocabulary development in children with WS, and underscores the need for more longitudinal, developmental trajectory analyses in defining social phenotypes in NDDs.

van der Fluit et al. (2012) explored relations between performance on a lab-based social perception and social cognition measure—the Social Attribution Task (SAT)—and parent reports of communication and reciprocal social skills in children with WS. They report significant correlations between parent-reported social reciprocity and the typicality of responses on the SAT after taking into account variability in intellectual functioning, suggesting a unique contribution of social cognition deficits to the social reciprocity difficulties of individuals with WS. These authors further analyzed children's responses on the SAT after receiving specific instructions regarding stimuli- interpretation, and revealed a facilitative effect of providing additional structure on social attributions, especially for the children with WS with higher intellectual functioning (e.g., improvements in the quality of the their narratives), finding that may have important implications for developing interventions targeting particular social functioning deficits in this population.

Martens et al. (2012) explored another type of social judgment—attribution of trustworthiness to human faces—in individuals with WS, who have been consistently described as overly friendly and driven to approach strangers. They used a fairly new methodology—computer mouse-tracking—to capture the continuous cognitive dynamics of social-evaluative judgments as they occur in real time. With this technique that allows to visually observe and quantify the competition between responses before the final approach/avoid decision is made, they demonstrated that the WS group showed an approach bias in their increased tendency to initially deviate toward untrustworthy faces, despite discriminating between mild and extreme degrees of trustworthiness in their final response, as the typical controls did. This methodology provides insights into the dynamics of cognitive processing underlying the hypersociability distinctive to the WS social-behavioral phenotype.

Another novel methodological approach to examining a fundamental aspect of social phenotypes—face expertise—was adopted by Parish-Morris et al. (2013). Using eye-tracking technology, these researchers investigated how visual attention to dynamic faces and objects related to face recognition considered on a continuum of ability, in a combined sample of children with ASD and TD controls. They found that visual attention to faces predicted face perception skill in the combined ASD and TD sample after accounting for the effect of age, but that gaze patterns did not vary significantly by diagnostic group. By taking a dimensional approach instead of focusing on group comparisons, these authors were able to capitalize on the heterogeneity of face processing abilities found in both ASD and TD children, and carried out a more direct test of the hypothesized link between social attention and face expertise.

In the final article, Järvinen et al. (2012) describe a multimodal approach to exploring sensitivity to social and non-social visually and aurally presented affective stimuli in individuals with WS and typical controls, as reflected in behavioral responses and autonomic arousal, measured by changes in electrodermal and heart rate activity. Analyses of the psychophysiological measures revealed significant differences in autonomic nervous system (ANS) sensitivity to different categories of affective stimuli and between the visual and auditory domains in individuals with WS—a pattern of ANS reactivity different from that of the typical controls. These findings show that analyses of ANS functioning provide a useful complementary perspective to behavioral paradigms, and a more direct avenue for understanding the neural substrates underlying distinctive patterns of responsiveness to social information found across different disorders.

To conclude, the articles presented in this e-book illustrate a variety of methodological approaches that have the potential to advance our understanding of how phenotypic outcomes emerge from the complex interplay of genetic constraints, environmental conditions and individual experiences, along atypical or typical developmental trajectories. These innovative approaches provide a glimpse into the fascinating new directions in which the study of NDDs may evolve. We hope you will share our enthusiasm for this research and its promises.
